# Post-Psychedelic Reductions in Experiential Avoidance Are Associated With Decreases in Depression Severity and Suicidal Ideation

**DOI:** 10.3389/fpsyt.2020.00782

**Published:** 2020-08-07

**Authors:** Richard J. Zeifman, Anne C. Wagner, Ros Watts, Hannes Kettner, Lea J. Mertens, Robin L. Carhart-Harris

**Affiliations:** ^1^Centre for Psychedelic Research, Department of Brain Sciences, Faculty of Medicine, Imperial College London, London, United Kingdom; ^2^Remedy, Toronto, ON, Canada; ^3^Department of Molecular Neuroimaging, Central Institute of Mental Health (CIMH), Medical Faculty Mannheim, University Heidelberg, Mannheim, Germany

**Keywords:** psychedelics, experiential avoidance, suicidal ideation, depression severity, transdiagnostic mechanisms

## Abstract

Psychedelic therapy shows promise as a novel intervention for a wide range of mental health concerns but its therapeutic action is incompletely understood. In line with acceptance and commitment therapy’s (ACT’s) transdiagnostic model, qualitative research has suggested that reductions in experiential avoidance are an important component of therapeutic outcomes associated with psychedelics. However, limited research has quantitatively explored the association between decreases in experiential avoidance and therapeutic outcomes associated with psychedelics. Therefore, in two prospective studies, using convenience samples of individuals with plans to use a psychedelic, we explored the impact of psychedelic use on experiential avoidance, depression severity, and suicidal ideation, as well as relationships between changes in these outcomes. Participants (Study 1, *N*=104; Study 2, *N*=254) completed self-report questionnaires of depression severity, suicidal ideation, and experiential avoidance: 1) before using a psychedelic (in ceremonial and non-ceremonial contexts), as well as 2) 2-weeks and 3) 4-weeks after psychedelic use. Across both studies, repeated measures ANOVAs indicated significant decreases in experiential avoidance, depression severity, and suicidal ideation after psychedelic use. Furthermore, decreases in experiential avoidance were significantly associated with decreases in depression severity and suicidal ideation. These results suggest that psychedelics may lead to significant decreases in experiential avoidance, depression severity, and suicidal ideation. Additionally, these findings imply that reduced experiential avoidance may be a transdiagnostic mechanism mediating treatment success within psychedelic therapy. We conclude that integrating psychedelics with psychotherapeutic interventions that target experiential avoidance (e.g. ACT) may enhance therapeutic outcomes.

## Introduction

“Afterwards, I allowed myself to experience everything—even if it is sadness. Now I know how to deal with my feelings rather than repress them” (1)

Serotonergic psychedelics, a class of pharmacological agents that act as serotonin 2A receptor (5-HT2AR) agonists, including psilocybin, lysergic acid diethylamide (LSD), and dimethyltryptamine (DMT; contained in the brew ayahuasca), are currently receiving attention within psychiatry and mental health as novel interventions for a wide range of mental health concerns [for reviews, see (2, 3)]. Randomized controlled trials and open-label trials have found promising results for psychedelic therapy in the treatment of distress associated with a life-threatening illness [e.g., (4–6)], substance use disorders [e.g., (7–10)], obsessive-compulsive disorder (11), depression [e.g., (12–14)], and suicidal ideation (13, 15).

Given the wide range of mental health concerns for which psychedelic therapy has shown promise, it has been suggested that this treatment approach may target underlying transdiagnostic mechanisms (16–21). Recent research has indicated that the intensity of “mystical-type” (4, 6, 7, 9, 22) or “peak” experiences engendered by psychedelics (23) is predictive of positive therapeutic outcomes; however, it remains unclear *how* (i.e., *via* what fundamental psychological and neurobiological mechanisms) psychedelic therapy brings about lasting therapeutic changes. Identifying such underlying mechanisms is important for a number of reasons, including that it might improve our overall understanding of psychopathology [e.g., see Research Domain Criteria (RDoC); (24)], optimize therapeutic outcomes, and choice of treatment for clients, as well as guide treatment development, refinement and delivery (25).

### Experiential Avoidance as a Potential Transdiagnostic Mechanism

One potential transdiagnostic mechanism that may account for the positive therapeutic outcomes associated with psychedelic therapy is reduced experiential avoidance [i.e., thoughts or behaviors that are intended to avoid or suppress aversive states; (26)]. The increasingly popular psychotherapeutic model termed “acceptance and commitment therapy” (ACT) identifies experiential avoidance as an essential component of psychological flexibility [i.e., openness to one’s experiences and engagement in behaviors that are congruent with one’s values; (26)] and as a transdiagnostically relevant factor that is central to the development and treatment of psychopathology (26). In line with this view, clinical and experimental research has begun to suggest that psychedelics may lead to decreases in experiential avoidance. For example, qualitative analysis of interviews conducted among individuals that received psilocybin therapy for treatment-resistant depression identified shifts from emotional avoidance to emotional acceptance as a central theme underlying therapeutic change (1). Similarly, within non-clinical samples, experimental research has found that administration of ayahuasca is associated with increases in experiential acceptance (27–30), which is inversely associated with experiential avoidance (31).

More recently, two studies (18, 32) examined the relationship between improved mental health following psychedelic use and decreases on the revised Acceptance and Action Questionnaire [AAQ-II; (33)], a frequently used measure of both experiential avoidance and psychological flexibility. Both studies found that psychedelic use was associated with post-psychedelic decreases in AAQ-II scores (18, 32). Furthermore, Close et al. (32) found that, following psychedelic use, decreased AAQ-II scores were associated with decreases in depression severity. Additionally, Davis et al. (18) found that decreased AAQ-II scores indirectly affected the relationship between acute psychedelic experiences (mystical-type experiences and psychological insight) and decreases in anxiety and depression severity. Importantly, however, extant research suggests that the AAQ-II shows poor discriminant validity from psychological distress (34–38). This research suggests that the AAQ-II does not measure the *process* of experiential avoidance itself and may be better conceptualized as an *outcome* related to psychological distress [e.g., one of the AAQ-II items is “Emotions cause problems in my life.”; (36)]. This is problematic given that the *process* of experiential avoidance has been conceptualized as distinct from the *outcome* of psychological distress (39). Furthermore, this distinction between *outcome* and *process* is especially important when examining whether reductions in experiential avoidance is a mechanism through which psychedelics lead to lasting therapeutic changes. Accordingly, there remains a need for additional research examining decreases in the *process* of experiential avoidance as a psychological mechanism through which psychedelics lead to positive therapeutic *outcomes*.

### Psychedelics, Depression, and Suicidal Ideation

Worldwide, approximately 350 million people struggle with depression (40) and many more struggle with subclinical depressive symptoms (41). Furthermore, suicide, which is a key concern linked to depression (42) that can also occur independent of it (43), accounts for nearly one million deaths per year (40). Moreover, within the United States, suicide rates have increased over the last two decades (44, 45).

Despite the burden associated with depression and suicide, there are significant limitations surrounding current first-line interventions [i.e., cognitive behavioral therapy and selective serotonin reuptake inhibitors (SSRIs)]. For instance, many individuals do not respond to treatment (46, 47) and, compared with placebo and control groups, effect sizes are modest (48–51). Furthermore, there is limited evidence for the efficacy of SSRIs for individuals with mild to moderate depression severity (52, 53), and questions exist about the specificity and robustness of their therapeutic action (54), their safety and side-effect profile (55–57), as well as delayed latency of therapeutic action, and complications concerning withdrawal (58–60). There is, therefore, an important need for exploring novel interventions for depression (61) and suicidal ideation (62).

Recent research has begun exploring psychedelic therapy as a novel intervention for depression and suicidal ideation. In a randomized controlled trial, among individuals with treatment-resistant major depressive disorder (MDD), administration of ayahuasca was associated with large effect sizes for decreases in depression severity (14) and suicidal ideation (15). Similarly, in an open-label trial, individuals with treatment-resistant MDD that received psilocybin therapy showed large decreases in depression severity and suicidal ideation (13). Additionally, among individuals with distress associated with a life-threatening illness, randomized controlled trials have indicated that psilocybin-assisted psychotherapy leads to long-lasting decreases in anxiety and depression severity (4, 6). Recent studies have also shown that psychedelics can lead to decreases in depression severity within non-clinical samples (63–66). Given SSRIs putative limited efficacy for mild to moderate depression (52) and suicidal ideation (51), as well as evidence favoring the principle of prophylactic intervention in mental healthcare (67, 68) there may be scope for assessing whether psychedelics can improve and protect mental health in non-clinical populations. Accordingly, there is a need for additional research on the impact of psychedelics on depression severity and suicidal ideation, as well as a greater understanding of the mechanisms through which psychedelics lead to positive therapeutic outcomes.

In line with ACT’s transdiagnostic model of psychopathology (26), one potential explanation for the impact of psychedelics on depression severity and suicidality is *via* decreases in experiential avoidance. Experiential avoidance is prospectively predictive of depression severity [e.g., (69, 70)] and suicidal ideation [e.g., (71, 72)]. Moreover, within psychotherapeutic treatments, decreases in experiential avoidance are associated with, and predictive of, subsequent decreases in depression severity (73, 74) and suicidal ideation (74, 75). However, there is limited research on whether decreases in the *process* of experiential avoidance occur in parallel with positive therapeutic *outcomes* (e.g., depression severity and suicidal ideation) after the use of psychedelics.

### Summary

In sum, despite research suggesting that psychedelics lead to improvements in mental health outcomes, there is currently little understanding of whether psychedelics lead to decreases in depression severity and suicidal ideation within non-clinical samples. Furthermore, while preliminary research suggests that reductions in experiential avoidance may play a key role in psychedelic therapy, there is currently limited research that has examined the association between decreases in experiential avoidance and positive therapeutic outcomes following psychedelic use. To address these knowledge gaps, the aims of the present study were: (a) to examine the impact of psychedelic use on experiential avoidance, depression severity, and suicidal ideation; (b) to examine whether reductions in experiential avoidance would be associated with reductions in depression severity and suicidal ideation following psychedelic use. We hypothesized that:

1. Psychedelic use will be associated with decreases in (a) experiential avoidance, (b) depression severity, and (c) suicidal ideation.
2. Decreases in experiential avoidance after psychedelic use will be associated with decreases in (a) depression severity and (b) suicidal ideation.

## Methods: Study 1

### Procedure and Participants

We conducted a prospective cohort study utilizing an online convenience sample of individuals with plans to use a psychedelic. Participants were recruited *via* online advertisements shared through social media (e.g., Facebook, Twitter), email newsletters, and online forums (e.g., Reddit). Participants reviewed information related to the study design online, provided informed consent, and their e-mail address. Based on when they planned to use a psychedelic, individuals were sent emails at three key time points [i.e., 1-week prior to psychedelic use (baseline), as well as 2-weeks and 4-weeks post-psychedelic use] reminding them to complete the online surveys. To be eligible to participate, participants were required to endorse: (a) being ≥18 years old, (b) comprehension of English, and (c) an intention to use a psychedelic (i.e., psilocybin/magic mushrooms/truffles, LSD/1P-LSD, ayahuasca, DMT/5-MeO-DMT, salvia divinorum, mescaline, or iboga/ibogaine). The study received approval from Imperial College London’s Imperial College Research Ethics Committee (ICREC) and the Joint Research Compliance Office (JRCO).

Participants were recruited from April 2018 to May 2019. A total of 279 individuals enrolled in the study. Individuals that failed to (a) respond to any of the outcome variables at baseline (*n*=53) or (b) respond to both follow-up surveys (*n*=97), were removed from all analyses. Additionally, four individuals reported plans to use substances that are not considered serotonergic psychedelics [i.e., ketamine, dextromethorphine, and 3,4-methylenedioxy-methamphetamine (MDMA)] and were removed from all analyses. Finally, individuals with a score of 0 on the Quick Inventory of Depressive Symptoms [QIDS; (76)] at baseline were excluded from all analyses (*n*=4). Given our interest in examining the effect of psychedelic use on the full spectrum of depression severity and research indicating that depression should be conceptualized as a dimensional rather than categorical construct [e.g., (77)], we included all individuals with QIDS > 0 at baseline in our final sample. The final sample included 104 individuals. For participant demographics, see **Table 1** (Study 1).

**Table 1:** Participant demographics (Study 1 and Study 2).

		Study 1	Study 2
Total Sample		104	254
Gender	Male Female Other	71 (68.3%) 31 (29.8%) 2 (1.9%)	137 (53.9%) 115 (45.3%) 2 (0.8%)
Age *M (SD)*		29.28 (*9.94*)	43.61 (*12.46*)
Education level	Did not graduate high school High school diploma/A-level education Bachelor’s degree (or equivalent) Post-graduate degree (e.g., MA, MD, PhD)	8 (8.3%) 42 (40.4%) 34 (31.5%) 20 (19.2%)	3 (1.2%) 20 (7.7%) 96 (37.8%) 135 (53.2%)
Employment status	Student Unemployed Part-time job Full-time job Retired	34 (32.7%) 8 (7.7%) 11 (11.5%) 47 (45.2%) 3 (2.9%)	26 (10.2%) 9 (3.5%) 47 (18.5%) 150 (59.1%) 22 (8.7%)
Nationality	United Kingdom United States Canada Germany Sweden Netherlands Other	25 (24.0%) 24 (23.1%) 11 (10.6%) 5 (4.8%) 6 (5.8%) 5 (4.8%) 28 (26.9%)	67 (26.4%) 90 (35.4%) 6 (2.4%) 8 (3.1%) 6 (2.4%) 8 (3.1%) 69 (27.2%)
Psychiatric history	Diagnosed with a psychiatric disorder Diagnosed with depression	48 (46.2%) 24 (23.1%)	79 (31.1%) 47 (18.5%)
Previous psychedelic use	Never Once 2–5 times 6–10 times 11–20 times More than 21 times	10 (9.6%) 3 (2.9%) 25 (24.0%) 23 (22.1%) 15 (14.4%) 28 (26.9%)	106 (41.7%) 23 (9.1%) 46 (18.1%) 23 (9.1%) 27 (10.6%) 29 (11.4%)

### Measures

#### Experiential Avoidance

Experiential avoidance was measured using the Brief Experiential Avoidance Questionnaire [BEAQ; (78)]. The BEAQ is a 15-item self-report measure including items such as “The key to a good life is never feeling any pain” and “I work hard to keep out upset feelings.” Participants rated the extent to which they agree with each item on a 6-point scale from 1 (strongly disagree) to 6 (strongly agree). One item is reverse-scored. Higher scores indicate higher levels of experiential avoidance. The BEAQ has been shown to have strong internal consistency and discriminant validity, including being more strongly correlated with measures of avoidance than with measures of psychological distress (36, 37, 79). The BEAQ was measured at baseline (α=.89), 2-weeks (α=.89), and 4-weeks (α=.90).

#### Depression Severity

Depression severity was measured using the Quick Inventory of Depressive Symptoms [QIDS; (76)]. The QIDS is a 16-item self-report measure that assesses the presence of the nine key diagnostically relevant symptoms of major depression (i.e., depressed mood, loss of interest or pleasure, concentration/decision-making difficulties, negative self-outlook, low energy/fatigability, sleep disturbance, weight/appetite change, psychomotor changes, and suicidal ideation) over the previous 7 days. Items are rated on a scale from 0 to 3, with higher scores indicating higher levels of depression severity (i.e., 0–5 no depression, 6–10 mild, 11–15 moderate, 16–20 severe, and 21–27 very severe). The QIDS has shown good internal consistency (76, 79). The QIDS was measured at baseline (α=.85), 2-weeks (α=.76), and 4-weeks (α=.75).

#### Suicidal Ideation

Suicidal ideation was measured using the Suicidal Ideation Attributes Scale [SIDAS; (80)] and the QIDS-suicidality item (QIDS-SI). The SIDAS is a 5-item self-report measure of suicidal ideation that assesses the frequency, controllability, closeness to attempt, level of distress, and impact on daily functioning associated with suicidal thoughts. Items are rated on a scale from 0 (e.g., never) to 10 (e.g., always), with one reverse scored item. Higher scores indicate higher levels of suicidal ideation. The SIDAS has been shown to have strong internal consistency and good convergent validity (80). The SIDAS was measured at baseline (α=.83), 2-weeks (α=.76), and 4-weeks (α=.82). The QIDS-SI is a single-item assessing suicidal ideation over the past 7 days. The QIDS-SI is rated on a scale from 0 to 3, with ratings as follows: 0 (“I do not think of suicide or death.”), 1 (“I feel that life is empty or wonder if it’s worth living.”), 2 (“I think of suicide or death several times a week for several minutes.”), and 3 (“I think of suicide or death several times a day in some detail, or I have made specific plans for suicide or have actually tried to take my life.”). The QIDS-SI was measured at baseline, 2-weeks, and 4-weeks.

### Analyses

Variables were examined for normality of distribution and were found to deviate from normality (see **Table 2**). Therefore, when appropriate, non-parametric tests were used. To increase power and minimize type II errors, in line with past research (81–84), we created a composite measure of suicidal ideation (SI_composite_) by summing Z-scores for the SIDAS and QIDS-SI.

**Table 2:** Study 1: Means, standard deviations, and correlation coefficients for baseline measures.

Measure	Mean (*SD*)	Skewness (*SE*)	Kurtosis (*SE*)	Correlation Coefficients			
				1.	2.	3.	4.
1. Experiential Avoidance (BEAQ)	44.12 (*13.90*)	0.46 (0*.24)*	0.12 (0*.47*)	–			
2. Depression Severity (QIDS)	7.92 (*5.34*)	0.88 (*0.24*)	0.04 (*0.47*)	.552***	–		
3. Suicidal Ideation (SI_composite_)	0.48 *(2.31)*	1.73 (0*.24*)	2.68 (*0.47*)	.408***	.636***	–	
4. Suicidal Ideation (SIDAS)	3.76 (*6.46*)	2.30 (0*.24*)	5.99 (*0.47*)	.414***	.590***	.948***	–
5. Suicidal Ideation (QIDS-SI)	0.54 (0*.85*)	1.44 (*0.24*)	1.08 (*0.47*)	.363***	.612***	.905***	.789***

N=104; Correlation coefficients are Spearman’s rho; ***p < .001.

BEAQ, Brief Experiential Avoidance Questionnaire; QIDS, Quick Inventory of Depressive Symptoms; SI_composite_, composite measure of suicidal ideation; SIDAS, Suicidal Ideation Attributes Scale; QIDS-SI, QIDS-suicidality item.

#### Hypothesis #1: Decreases in Experiential Avoidance, Depression Severity, and Suicidal Ideation Over Time

To examine whether there were decreases in (a) experiential avoidance (BEAQ), (b) depression severity (QIDS), and (c) suicidal ideation (SI_composite_) after psychedelic use, we conducted three general linear models (GLM) repeated measures ANOVAs, which are robust to non-parametric data with large samples [i.e., > 30; (85, 86)], with Bonferroni corrections for multiple comparisons. We also calculated Cohen’s *d* effect sizes for decreases in experiential avoidance, depression severity, and suicidal ideation over time.

#### Hypothesis # 2: Association Between Decreases in Experiential Avoidance, Depression Severity, and Suicidal Ideation

To determine whether decreases in experiential avoidance were associated with decreases in (a) depression severity and (b) suicidal ideation, we calculated change scores (time point − baseline) for experiential avoidance (BEAQ), depression severity (QIDS), and suicidal ideation (SI_composite_). Next, we conducted correlation analyses using Spearman’s rho (due to data violating assumptions of normality).

All analyses were conducted using the full sample (*N*=104) and in the subsample of individuals with mild to very severe depression severity (i.e., QIDS ≥ 6; *n*=60) at baseline. Analyses were conducted using IBM SPSS Statistics (Version 25). For all analyses, we set the two-tailed alpha level at .05.

## Results: Study 1

### Descriptive Statistics

Participants reported planning to use the following psychedelics: psilocybin/magic mushrooms/truffles (*n*=46; 44.2%), LSD/1P-LSD (*n*=46; 44.2%), ayahuasca (*n*=2; 1.9%), DMT (*n*=5; 4.8%), 5-MeO-DMT (*n*=1; 1.0%), 4-AcO-DMT (*n*=1; 1.0%), 5-MeO-MiPT (*n*=1; 1.0%), more than one psychedelic (i.e., LSD, DMT, and psilocybin; *n*=1; 1.0%), and either psilocybin or LSD (*n*=1; 1.0%). For means, standards deviations, and correlation coefficients for baseline measures, see **Table 2**.

### Primary Analyses

#### Hypothesis 1a: Decreases in Experiential Avoidance Over Time

We examined whether there were significant decreases in experiential avoidance (BEAQ) over time (measured at baseline, 2-weeks, and 4-weeks).

**Figure 1:**
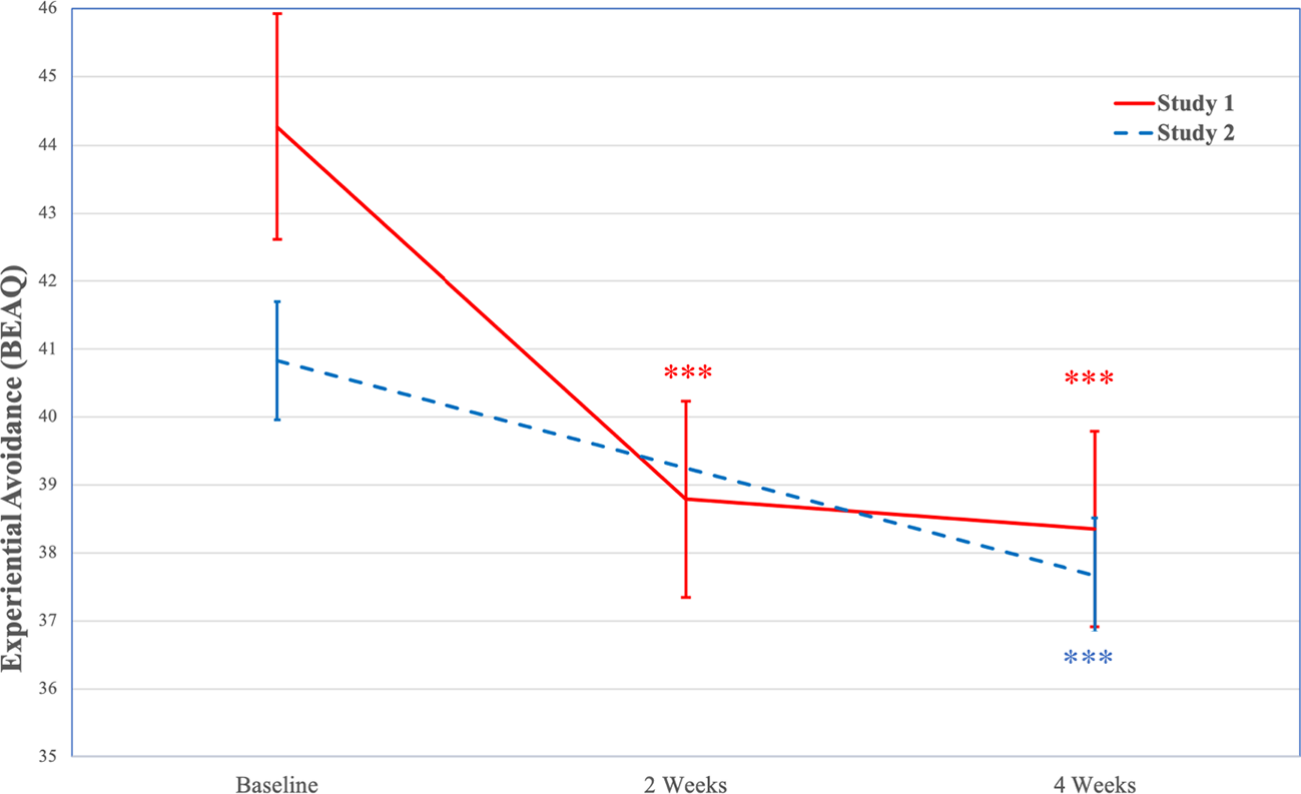
Decreases in Experiential Avoidance (BEAQ) Over Time (Study 1 and Study 2). Note. Study 1 *N*=104; Study 2 *N*=254; BEAQ is only assessed at baseline and 4-weeks in Study 2; ***p < .001. BEAQ, Brief Experiential Avoidance Questionnaire.

In the full sample, Mauchly’s test indicated that the assumption of sphericity had been violated χ2(2)=36.52, *p*<.001, therefore degrees of freedom were corrected using Huynh-Feldt estimates of sphericity (ϵ=0.72). A GLM repeated measures ANOVA indicated significant decreases in experiential avoidance over time, *F*(1.46, 107.72)=20.36, *p*<.001. Post hoc tests using the Bonferroni correction indicated experiential avoidance decreased significantly from baseline (*M*=44.27, *SE*=1.65) to 2-weeks (*M*=38.79, *SE*=1.44, *p*<.001; *d*=0.88) and 4-weeks (*M*=38.35, *SE*=1.44, *p*<.001; *d*=1.07), with no significant changes from 2-weeks to 4-weeks (*p*=1.00; *d*=0.17). See **Figure 1** for changes in experiential avoidance (BEAQ) over time.

Among individuals with mild to very severe depression severity, Mauchly’s test indicated that the assumption of sphericity had been violated χ2=31.96, *p*<.001, therefore degrees of freedom were corrected using Huynh-Feldt estimates of sphericity (ϵ=0.67). A GLM repeated measures ANOVA indicated significant decreases in experiential avoidance over time, *F*(1.34, 60.44)=22.82, *p*<.001. Post hoc tests using the Bonferroni correction indicated experiential avoidance decreased significantly from baseline (*M*=50.24, *SE*=2.00) to 2-weeks (*M*=41.71, *SE*=1.97, *p*<.001; *d*=1.53) and 4-weeks (*M*=41.74, *SE*=1.96, *p*<.001; *d*=1.46), with no significant changes from 2-weeks to 4-weeks (*p*=1.00; *d*=0.01).

#### Hypothesis 1b: Decreases in Depression Severity Over Time

We examined whether there were significant decreases in depression severity (QIDS) over time (measured at baseline, 2-weeks, and 4-weeks).

**Figure 2:**
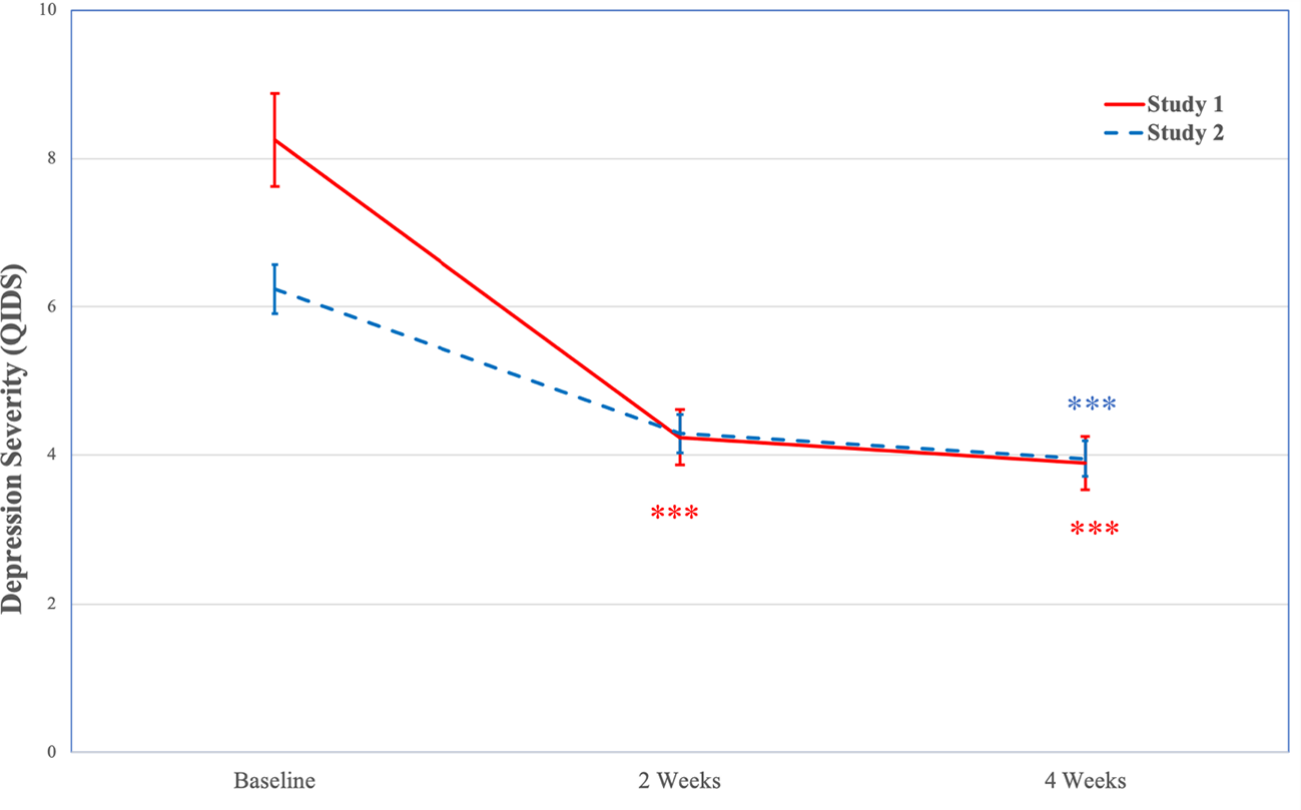
Decreases in Depression Severity (QIDS) Over Time (Study 1 and Study 2). Note. Study 1 *N*=104; Study 2 *N*=254; ***p < .001.QIDS, Quick Inventory of Depressive Symptoms.

In the full sample, Mauchly’s test indicated that the assumption of sphericity had been violated χ2=66.79, *p*<.001, therefore degrees of freedom were corrected using Huynh-Feldt estimates of sphericity (ϵ=0.63). A GLM repeated measures ANOVA indicated significant decreases in depression severity over time, *F*(1.26, 93.36)=47.13, *p*<.001. Post hoc tests using the Bonferroni correction indicated that depression severity decreased significantly from baseline (*M*=8.25, *SE*=0.63) to 2-weeks (*M*=4.24, *SE*=0.37, *p*<.001; *d*=1.41) and 4-weeks (*M*=3.89, *SE*=0.36, *p*<.001; *d*=1.60), with no significant changes from 2-weeks to 4-weeks (*p*=.446; *d*=0.34). See **Figure 2** for changes in depression severity (QIDS) over time.

Among individuals with mild to very severe depression severity, Mauchly’s test indicated that the assumption of sphericity had been violated χ2(2)=32.17, *p*<.001, therefore degrees of freedom were corrected using Huynh-Feldt estimates of sphericity (ϵ=0.67). A GLM repeated measures ANOVA indicated significant decreases in depression severity over time, *F*(1.34, 60.35)=61.65, *p*<.001. Post hoc tests using the Bonferroni correction indicated that depression severity decreased significantly from baseline (*M*=11.37, *SE*=0.69) to 2-weeks (*M*=5.22, *SE*=0.53, *p*<.001; *d*=2.33) and 4-weeks (*M*=4.70, *SE*=0.51, *p*<.001; *d*=2.58), with no significant changes from 2-weeks to 4-weeks (*p*=.443; *d*=0.44).

#### Hypothesis 1c: Decreases in Suicidal Ideation Over Time

We examined whether there were significant changes in suicidal ideation (SI_composite_) over time (measured at baseline, 2-weeks, and 4-weeks).

**Figure 3:**
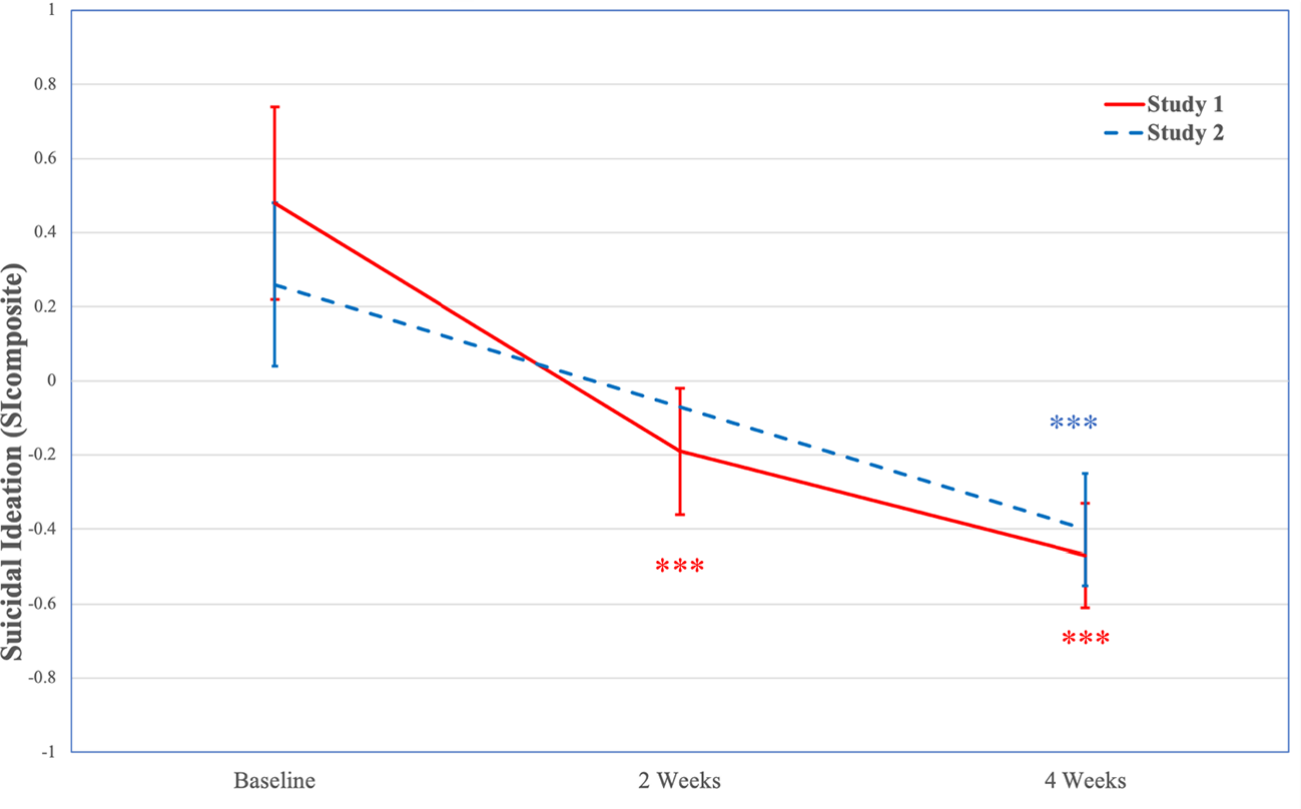
Decreases in Suicidal Ideation (SI_composite_) Over Time (Study 1 and Study 2). Note. Study 1 *N*=104; Study 2 *N*=254; SI_composite_ is only assessed at baseline and 4-weeks in Study 2; ***p < .001. SI_composite_, composite measure of suicidal ideation.

In the full sample, Mauchly’s test indicated that the assumption of sphericity had been violated χ2(2)=73.00, *p*<.001, therefore degrees of freedom were corrected using Huynh-Feldt estimates of sphericity (ϵ=0.63). A GLM repeated measures ANOVA indicated significant decreases in suicidal ideation (SI_composite_) over time, *F*(1.25, 97.54)=16.20, *p*<.001. Post hoc tests using the Bonferroni correction indicated suicidal ideation (SI_composite_) decreased significantly from baseline (*M*=0.48, *SE*=0.26) to 2-weeks (*M*=−0.19, *SE*=0.17, *p*<.001; *d*=0.86) and 4-weeks (*M*=−0.47, *SE*=0.14, *p*<.001; *d*=0.98), with significant decreases from 2-weeks to 4-weeks (*p*=.003; *d*=0.69). See **Figure 3** for changes in suicidal ideation (SI_composite_) over time.

Among individuals with mild to very severe depression severity, Mauchly’s test indicated that the assumption of sphericity had been violated χ2(2)=37.41, *p*<.001, therefore degrees of freedom were corrected using Huynh-Feldt estimates of sphericity (ϵ=0.65). A GLM repeated measures ANOVA indicated significant decreases in suicidal ideation (SI_composite_) over time, *F*(1.29, 58.14)=17.47, *p*<.001. Post hoc tests using the Bonferroni correction indicated suicidal ideation (SI_composite_) decreased significantly from baseline (*M*=0.69, *SE*=0.32) to 2-weeks (*M*=−0.27, *SE*=0.22, *p*<.001; *d*=1.05) and 4-weeks (*M*=−0.64, *SE*=0.19, *p*=.007; *d*=1.22), with significant decreases from 2-weeks to 4-weeks (*p*=.007; *d*=0.84).

#### Hypothesis 2a: Association Between Decreases in Experiential Avoidance and Depression Severity

In the full sample, results indicated significant associations between changes in experiential avoidance (BEAQ) and changes in depression severity (QIDS) at 2-weeks (Spearman’s rho=.371, *p* <.001) and 4-weeks (Spearman’s rho=.516, *p<*.001).

Among individuals with mild to very severe depression severity, results indicated significant associations between decreases in experiential avoidance (BEAQ) and decreases in depression severity (QIDS) at 2-weeks (Spearman’s rho=.322, *p* =.014) and 4-weeks (Spearman’s rho=.486, *p<*.001).

#### Hypothesis 2b: Association Between Decreases Experiential Avoidance and Suicidal Ideation

In the full sample, results indicated significant associations between changes in experiential avoidance (BEAQ) and changes in suicidal ideation (SI_composite_) at 2-weeks (Spearman’s rho=.371, *p*<.001) and 4-weeks (Spearman’s rho=.461, *p*<.001).

Among individuals with mild to very severe depression severity, results indicated significant associations between decreases experiential avoidance (BEAQ) and decreases in suicidal ideation (SI_composite_) at 2-weeks (Spearman’s rho=.361, *p*=.005) and 4-weeks (Spearman’s rho=.423, *p*=.003).

## Methods: Study 2

### Procedures and Participants

To supplement Study 1 with data from a second independent study and sample, we conducted a separate prospective cohort study but this time designed and advertised for individuals attending psychedelic ceremonies. Such ceremonies usually involve the presence of one or more “facilitators”*—*individuals who aim to provide a safe, conducive environment, emotional support, as well as certain contextual stimuli which are intended to enhance or structure the psychedelic experience. Most often these contextual elements include recorded, or live music, decorative and ritualistic objects, and restriction of sensory input through dim lighting or the provision of eyeshades. Retreat centers and individuals offering psychedelic ceremonies were contacted by the research team and described the design of the study. Online advertisements raised the visibility of the surveys for individuals planning to attend such ceremonies. Interested participants reviewed information related to the study design, as well as provided informed consent and their e-mail address. Based on the time that they indicated planning to use a psychedelic, individuals were sent emails at various time points [e.g., within the 2-weeks prior to psychedelic use (baseline), as well as 2-weeks and 4-weeks after psychedelic use] reminding them to complete the relevant online surveys. To be eligible to participate, participants were required to endorse: (a) being ≥ 18 years old, (b) comprehension of English, and (c) intention to use a psychedelic (i.e., psilocybin/magic mushrooms/truffles, LSD/1P-LSD, ayahuasca, DMT/5-MeO-DMT, salvia divinorum, mescaline, or iboga/ibogaine) within a ceremonial setting. The study received its own approval from Imperial College London’s Imperial College Research Ethics Committee (ICREC) and the Joint Research Compliance Office (JRCO), independent of study one.

Participants were recruited from April 2018 to June 2019. A total of 478 individuals enrolled in the study. Individuals that failed to (a) respond to any of the outcome variables at baseline (*n*=32) or (b) respond to both of the follow-up surveys (*n*=206) were removed from all analyses. Additionally, two individuals reported planning to use kambo, which is not a serotonergic psychedelic and were, therefore, removed from all analyses. Finally, similar to Study 1, individuals with a score of 0 on the QIDS at baseline were excluded from all analyses (*n*=5). The final sample included 254 individuals. For participant demographics, see **Table 1** (Study 2).

### Measures

Similar to Study 1, we used the BEAQ to measure experiential avoidance (baseline α=.86, 4-weeks α=.88), the QIDS to measure depression severity (baseline α=.78; 2-weeks α=.68; 4-weeks α=.73), as well as the SIDAS (baseline α=.82; 4-weeks α=.70) and QIDS-SI to measure suicidal ideation (baseline, 2-weeks, and 4-weeks). In contrast to Study 1, the BEAQ and SIDAS were only measured at baseline and 4-weeks.

### Analyses

Variables were examined for normality of distribution and were found to deviate from normality (see **Table 3**). Therefore, when appropriate, non-parametric tests were used. Analyses conducted in Study 2 were identical to those conducted in Study 1, except that the BEAQ and SIDAS were not measured at 2-weeks. Therefore, the BEAQ and SI_composite_ are not included in analyses for that time point. All analyses were conducted using the full sample (*N*=254) and in the subsample of individuals with mild to very severe depression severity (i.e., QIDS ≥ 6; *n*=121) at baseline.

**Table 3:** Study 2: Means, standard deviations, and correlation coefficients for baseline measures.

Measure	Mean (*SD*)	Skewness (*SE*)	Kurtosis (*SE*)	Correlation Coefficients			
				1.	2.	3.	4.
1. Experiential Avoidance (BEAQ)	41.30 (*11.94*)	0.40 (0*.15*)	-0.21 (*0.30*)	–			
2. Depression Severity (QIDS)	6.46 (*4.34*)	1.27 (*0.15*)	1.63 (*0.30*)	.404***	–		
3. Suicidal Ideation (SI_composite_)	0.21 (2.05)	3.12 (.15)	12.66 *(0.30)*	.228***	.525***	–	
4. Suicidal Ideation (SIDAS)	2.21 (*5.20*)	4.00 (0*.15*)	22.02 (*0.30*)	.175**	.462***	.928***	–
5. Suicidal Ideation (QIDS-SI)	0.29 (*.64*)	2.35 (*0.15*)	5.15 (*0.30*)	.229***	.546***	.797***	.613***

N=254; Correlation coefficients are Spearman’s rho; **p < .01, ***p < .001.

BEAQ, Brief Experiential Avoidance Questionnaire; QIDS, Quick Inventory of Depressive Symptoms; SI_composite_, composite measure of suicidal ideation; SIDAS, Suicidal Ideation Attributes Scale; QIDS-SI, QIDS-suicidality item.

## Results: Study 2

### Descriptive Statistics

Participants reported planning to use the following psychedelics: psilocybin/magic mushrooms/truffles (*n*=199; 78.3%), ayahuasca/yage or an ayahuasca analog (i.e., peganum harmala + mimosa hostilis; *n*=48; 18.9%), San Pedro (*n*=2; 0.8%), and DMT (*n*=1; 0.4%). Four individuals (1.6%) reporting planning to use more than one psychedelic. For means, standards deviations, and correlation coefficients for baseline measures, see **Table 3**.

### Primary Analyses

#### Hypothesis 1a: Decreases in Experiential Avoidance Over Time

We examined whether there were significant decreases in experiential avoidance (BEAQ) over time (measured at baseline and 4-weeks).

In the full sample, a GLM repeated measures ANOVA indicated significant decreases in experiential avoidance from baseline (*M*=40.83, *SE*=0.87) to 4-weeks (*M*=37.67, *SE*=0.84), *F*(1, 191)=24.63, *p*<.001, with a moderate effect size (*d*=0.72). See **Figure 1** for changes in experiential avoidance (BEAQ) over time.

Among individuals with mild to very severe depression severity, a GLM repeated measures ANOVA indicated significant decreases in experiential avoidance from baseline (*M*=45.27, *SE*=1.33) to 4-weeks (*M*=41.01, *SE*=1.36), *F*(1, 88)=20.31, *p*<.001, with a large effect size (*d*=0.96).

#### Hypothesis 1b: Decreases in Depression Severity Over Time

We examined whether there were significant decreases in depression severity (QIDS) over time (measured at baseline, 2-weeks, and 4-weeks).

In the full sample, Mauchly’s test indicated that the assumption of sphericity had been violated χ2(2)=31.64, *p*<.001; therefore, degrees of freedom were corrected using Huynh-Feldt estimates of sphericity (ϵ=0.86). A GLM repeated measures ANOVA indicated significant decreases in depression severity over time, *F*(1.72, 259.48)=33.13, *p*<.001. Post hoc tests using the Bonferroni correction indicated that depression severity decreased significantly from baseline (*M*=6.24, *SE*=0.33) to 2-weeks (*M*=4.29, *SE*=0.26, *p*<.001; *d*=0.97) and 4-weeks (*M*=3.95, *SE*=0.24, *p*<.001; *d*=1.07), with no significant changes from 2-weeks to 4-weeks (*p*=.457; *d*=0.23). See **Figure 2** for changes in depression severity (QIDS) over time.

Among individuals with mild to very severe depression severity, Mauchly’s test indicated that the assumption of sphericity had been violated χ2(2)=16.50, *p*<.001; therefore, degrees of freedom were corrected using Huynh-Feldt estimates of sphericity (ϵ=0.85). A GLM repeated measures ANOVA indicated significant decreases in depression severity over time, *F*(1.69, 123.65)=56.40, *p*<.001. Post hoc tests using the Bonferroni correction indicated that depression severity decreased significantly from baseline (*M*=9.82, *SE*=0.45) to 2-weeks (*M*=5.37, *SE*=0.45, *p*<.001; *d*=1.82) and 4-weeks (*M*=5.11, *SE*=0.42, *p*<.001; *d*=2.09), with no significant changes from 2-weeks to 4-weeks (*p*=1.00; *d*=0.16).

#### Hypothesis 1c: Decreases in Suicidal Ideation Over Time

We examined whether there were significant decreases in suicidal ideation (SI_composite_) over time (measured at baseline and 4-weeks).

In the full sample, a GLM repeated measures ANOVA indicated significant decreases in suicidal ideation (SI_composite_) from baseline (*M*=0.16, *SE*=0.15) to 4-weeks (*M*=−0.28, *SE*=0.10), *F*(1, 191)=12.89, *p*<.001, with a moderate effect size (*d*=0.52). See **Figure 3** for changes in suicidal ideation (SI_composite_) over time.

Among individuals with mild to very severe depression severity, a GLM repeated measures ANOVA indicated significant decreases in suicidal ideation (SI_composite_) from baseline (*M*=0.26, *SE*=0.22) to 4-weeks (*M*=−0.40, *SE*=0.16), *F*(1, 88)=13.13, *p*<.001, with a moderate effect size (*d*=0.77).

#### Hypothesis 2a: Association Between Decreases in Experiential Avoidance and Depression Severity

In the full sample, results indicated a significant association between changes in experiential avoidance and changes in depression severity at 4-weeks (Spearman’s rho=.325, *p*<.001).

Among individuals with mild to very severe depression severity, results indicated a significant association between decreases in experiential avoidance and decreases in depression severity at 4-weeks (Spearman’s rho=.431, *p*<.001).

#### Hypothesis 2b: Association Between Decreases in Experiential Avoidance and Suicidal Ideation

In the full sample, results indicated a significant association between changes in experiential avoidance and changes in suicidal ideation (SI_composite_) at 4-weeks (Spearman’s rho=.154, *p*=.033).

Among individuals with mild to very severe depression severity, results indicated a significant association between decreases in experiential avoidance and decreases in suicidal ideation (SI_composite_) at 4-weeks (Spearman’s rho=.213, *p*=.045).

## Discussion

Previous research has indicated that psychedelics hold promise for the treatment of a wide range of psychiatric concerns; however, little research has been done to assess the impact of psychedelics on depression severity or suicidal ideation among non-clinical samples. Across two separate studies, we found significant decreases in depression severity and suicidal ideation 4-weeks after psychedelic use. Importantly, we did not find significant increases in depression severity or suicidal ideation from 2-weeks to 4-weeks, suggesting that the positive effects of psychedelics were reliably sustained for at least 1-month post-psychedelic use. These results are in line with clinical studies indicating that psychedelics can lead to decreases in depression severity [e.g., (13, 14)] and suicidal ideation (13, 15) and that these effects are sustained over time [e.g., (4, 6, 13)]. Importantly, in both studies, the majority of the participants (Study 1 = 72.2%; Study 2 = 81.9%) exhibited subclinical levels of baseline depression severity. Therefore, our results provide additional support for the possibility that the effects of psychedelics extend along the full spectrum of depression severity, from the severe [e.g., (13, 14)] to the mild end of the spectrum [e.g., (63–65)]. Furthermore, in light of previous research showing that first-line interventions for depression lead to minimal to no decreases in suicidal ideation (48, 51), it is promising and highly relevant to observe reductions in suicidal ideation here, in relation to psychedelic use.

### Exploring the Role of Experiential Avoidance

Despite growing evidence to support the therapeutic potential of psychedelics for treating a wide range of mental health concerns, there remains a limited understanding of the underlying psychological mechanisms that account for their therapeutic effects. In line with ACT’s transdiagnostic model of psychopathology (26), our aims included exploring the impact of psychedelic use on experiential avoidance and whether decreases in experiential avoidance were associated with decreases in depression severity and suicidal ideation. We found that use of psychedelics was associated with decreases in experiential avoidance 2-weeks later and was sustained for at least 4-weeks. These results are in line with past research indicating that administration of ayahuasca leads to increases in experiential *acceptance* (27–30) and that psychedelic use is associated with decreases in experiential avoidance (18, 32). Our results add evidence to the view that psychedelics can target putative transdiagnostic mechanisms underlying psychopathology. Based on these results, we suggest that psychedelics may show promise for the treatment of mental health concerns characterized by experiential avoidance [see (87)].

We also found that decreases in experiential avoidance were significantly associated with decreases in depression severity and suicidal ideation. These results are in line with the finding that moving from avoidance to acceptance was a key theme associated with therapeutic change among individuals with treatment-resistant depression that received psilocybin therapy as part of an open-label clinical trial (1). Relatedly, within this trial, our group found that psilocybin therapy was associated with increases in amygdala responsiveness (i.e., interpreted as an increase in emotional responsiveness) 1-day post-treatment, which were predictive of decreases in depression severity 5-weeks later (88). In the same sample, we also observed improvements in behavioral indices of emotional processing post psilocybin therapy (89), as well as a correction to the pessimism bias that is known to characterize severe depression (90).

We interpret these findings as consistent with a previous suggestion (91) that psychedelics enhance emotion-focused coping [i.e., actively dealing with or changing one’s relationship with negative emotions and their sources; (92)]. As one patient from our previous psilocybin trial noted: “*Afterwards, I allowed myself to experience everything—even if it is sadness. Now I know how to deal with my feelings rather than repress them*” (1). As such, moving from experiential avoidance to acceptance may play a key role in therapeutic outcomes associated with psychedelics. Therefore, we suggest that integrating psychedelics and psychotherapeutic interventions that specifically target experiential avoidance may help to enhance and prolong (93) the effectiveness of psychedelic therapy [for discussions, see (87, 94, 95)]. Experiential avoidance is a primary target of ACT (26), an evidence-based psychotherapeutic intervention for a range of mental health problems [for a meta-analysis, see (96)]. Accordingly, we are currently conducting a trial (clinicaltrials.gov, NCT 03429075) in which we are integrating components of ACT with psilocybin for major depression [see (94); also, see (97)].

### Limitations and Future Research

The findings of the present study should be considered in light of its limitations. First, this was an uncontrolled study lacking a control group or randomization procedure. As such, results may have been driven by factors other than psychedelic use, such as the mere passage of time, a completer/attrition bias*—*whereby individuals who responded negatively to the psychedelic dropped out of follow-up surveys, expectancy effects, and other biases (e.g., confirmation bias) may have also contributed to outcomes. Additionally, our study did not include data quality checks (e.g., to ensure participants were responding attentively to the survey). Future studies employing a similar design may therefore seek to improve attrition rates, collect information on expectation and drop-outs, and include data quality checks to address potential biases and confounds. Furthermore, given the essential role of context (i.e., set and setting) in relation to the effects of psychedelics (98), further research is needed to assess the role that contextual variables (e.g., intentions, dosage, duration of the ceremony) may play in mediating outcomes.

As touched on above, given that our sample was a convenience sample of individuals with interest in using psychedelics and that it relied on their self-reports, it may be vulnerable to expectancy effects and demand characteristics. Therefore, further work is required to assess whether the present findings translate to clinical populations and controlled studies in which participants are randomized to receive a psychedelic, a placebo, or another active treatment. Future research would also benefit from including clinician-assessed depression severity and suicidal ideation, as well as behavioral measurement of experiential avoidance. Moreover, our results do not indicate whether the impact of psychedelic use on experiential avoidance, depression severity, and suicidal ideation were sustained beyond 4-weeks after psychedelic use. Therefore, future research would benefit from including a longer follow-up period. Additionally, while we found a significant relationship between decreases in experiential avoidance and decreases in depression severity and suicidal ideation following psychedelic use, these associations were small to moderate (Spearman’s rho = .154–.516). Accordingly, there is a need to identify additional psychological mechanisms that may account for positive therapeutic outcomes associated with psychedelic use, such as emotional breakthrough [e.g., (99)], insight [e.g., (18)], decentering [e.g., (29)], and connectedness [e.g., (100)]. Additionally, this study examined effects associated with use of a broad range of serotonergic psychedelics. Therefore, to increase specificity, future research examining the effects of specific serotonergic psychedelics will be necessary. The study of psychoactive drugs beyond just serotonergic psychedelics (e.g., ketamine and cannabis) would also be interesting to explore in future work.

Consistent with RDoC (24) recommendations, a focus on better defining the neurobiological basis of experiential avoidance and related phenomena would help develop the validity of the constructs. Indeed, it is natural to see increases in cognitive flexibility*—*as well as related ACT exercises such as “cognitive defusion”*—*as consistent with an acute de-weighting and subsequent revision of pathologically “over-weighted” internal predictive models (i.e., assumptions or beliefs). As was recently hypothesized by the (predictive-coding inspired) RElaxed Beliefs Under pSychedelics (REBUS) model of the therapeutic action of psychedelics (17).

In conclusion, consistent with prior hypotheses, we found that psychedelic use in ceremonial and non-ceremonial settings was associated with decreases in depression severity, suicidal ideation, and experiential avoidance; and decreases in experiential avoidance were associated with decreases in both depression severity and suicidal ideation. Overall, these results suggest that experiential avoidance may be a key transdiagnostic mechanism underlying therapeutic outcomes associated with psychedelics.

## Data Availability Statement

The datasets generated for this study are available on request to the corresponding author.

## Ethics Statement

The studies involving human participants were reviewed and approved by Imperial College Research Ethics Committee (ICREC), Imperial College London Joint Research Compliance Office (JRCO), Imperial College London. The patients/participants provided their written informed consent to participate in this study.

## Author Contributions

RC-H, LM, HK, and RW contributed to study design and conception. RZ, HK, and RC-H analyzed data and interpreted the results. RZ was responsible for the first draft of the manuscript. All authors contributed to the article and approved the submitted version.

## Funding

RZ received funding from the Canadian Institutes of Health Research. RC-H is supported by the Alex Mosley Charitable Trust and the Centre for Psychedelic Research was Founded by Tim Ferriss, Ad Astra Chandaria Foundation, Alexander and Bohdana Tamas, and the Nikean Foundation.

## Conflict of Interest

The authors declare that the research was conducted in the absence of any commercial or financial relationships that could be construed as a potential conflict of interest.
